# Sirtuin-1 isoforms differentially regulate mitochondrial function

**DOI:** 10.1093/geroni/igab046.2518

**Published:** 2021-12-17

**Authors:** Xiaomin Zhang, Fathima Ameer, Jasmine Crane, Gohar Azhar, Jeanne Wei

**Affiliations:** 1 University of Arkansas for Medical Sciences, Little Rock, Arkansas, United States; 2 UAMS, UAMS, Arkansas, United States

## Abstract

Alternative splicing generates multiple distinct isoforms that increase transcriptome and proteome diversity. Alternatively spliced isoforms may lose part of the protein domain and have different intracellular localization as well as distinct functions. The main form of the SIRT1 (SIRT1v1) protein contains 11 exons. We have identified two new isoforms, SIRT1v2 (lost 2 exons), and SIRT1v3 (lost 3 exons), but their effect on mitochondrial gene expression has not been reported. To study the effect of the three SIRT1 isoforms on mitochondrial gene expression and function, neuronal cells were transfected with SIRT1 isoforms v1, v2 or v3 plasmids, respectively. Gene expression was measured by quantitative reverse transcription PCR (RT-qPCR). Our data showed SIRT1 isoforms v1, v2 and v3 differentially regulated PCG-1alpha and PCG-1beta, which are the upstream regulators of mitochondrial structure and function. SIRT1v1 upregulated mitofusin-1 (MFN1), the mitochondrial dynamin-like GTPase (OPA1) gene, and the transcription factor A mitochondrial (TFAM) gene. In contrast, the SIRT1-v2 isoform repressed the MFN1, MFN2, and TFAM genes, while the SIRT1-v3 isoform repressed the MFN1 gene. In addition, the three SIRT1 isoforms differentially affected the mitochondrial respiratory complex I genes, including NDUFAB1, NDUFS1, NDUFV1, NDUFV2. The data indicates that SIRT1 regulates mitochondrial biogenesis and function through a signaling pathway involving PGC-1alpha, PCG-1beta, mitofusin 1 and 2, OPA1, and TFAM genes. Taken together, alternative splicing generated three SIRT1 isoform proteins with diverse functions. Age-related changes in the alternative splicing events are likely to impact sirtuin-regulated cellular functions and signaling pathways in aging and senescence.

